# Bromidotricarbon­yl[2-(pyridin-2-yl-κ*N*)-5-*p*-tolyl-1,3,4-oxadiazole-κ*N* ^3^]rhenium(I) dichloro­methane monosolvate

**DOI:** 10.1107/S160053681004969X

**Published:** 2010-12-04

**Authors:** Lin-Fang Shi, Yan-wei Li, Zhen-jun Si, Ying Guan, Hua-ru Cao

**Affiliations:** a College of Sciences, Zhejiang A&F University, Lin’an, Hangzhou, Zhejiang 311300, People’s Republic of China; b School of Chemical Engineering & Technology, Harbin Institute of Technology, Harbin 150001, People’s Republic of China; c School of Materials Science and Engineering, Changchun University of Science and Technology, Changchun 130000, People’s Republic of China

## Abstract

In the title compound, [ReBr(C_14_H_11_N_3_O)(CO)_3_]·CH_2_Cl_2_, the coordination geometry of the Re^I^ atom is a distorted ReC_3_N_2_Br octa­hedron with the carbonyl C atoms in a *fac* arrangement. Within the 2-(pyridin-2-yl)-5-*p*-tolyl-1,3,4-oxadiazole ligand, the dihedral angles between the oxadiazole ring and the pyridine (py) and benzene (bz) rings are 1.7 (2) and 7.1 (2)°, respectively, and the dihedral angle between the py and bz rings is 5.5 (2)°. In the crystal, aromatic π–π stacking between the oxadiazole rings of adjacent mol­ecules [centroid–centroid separation = 3.465 (3) Å] is seen.

## Related literature

For backgroud to phospho­resence in Re(I) complexes, see: Ley *et al.* (1997 ▶); Zhang *et al.* (2009 ▶). For a related structure, see: Rajendran *et al.* (2000 ▶). For the synthesis of the ligand, see: Demko & Sharpless (2001 ▶); Tamoto *et al.* (1997 ▶).
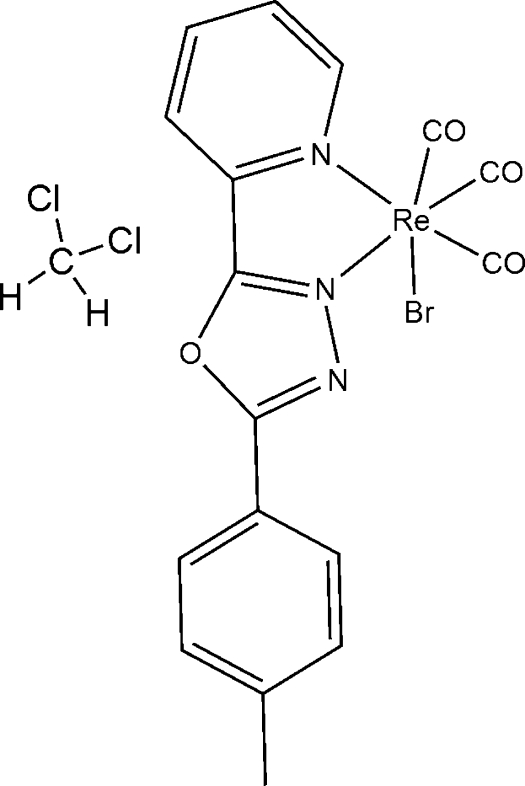


## Experimental

### 

#### Crystal data

[ReBr(C_14_H_11_N_3_O)(CO)_3_]·CH_2_Cl_2_
*M* *_r_* = 672.32
Monoclinic, 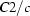

*a* = 12.590 (3) Å
*b* = 19.621 (4) Å
*c* = 17.660 (4) Å
β = 101.77 (3)°
*V* = 4270.8 (15) Å^3^
*Z* = 8
Mo *K*α radiation
μ = 7.84 mm^−1^
*T* = 293 K
0.25 × 0.22 × 0.11 mm

#### Data collection

Bruker SMART CCD diffractometer
Absorption correction: multi-scan (*SADABS*; Bruker, 2002) ▶ *T*_min_ = 0.149, *T*_max_ = 0.409
20653 measured reflections
4847 independent reflections
4254 reflections with *I* > 2σ(*I*)
*R*_int_ = 0.063

#### Refinement

*R*[*F* ^2^ > 2σ(*F* ^2^)] = 0.040
*wR*(*F* ^2^) = 0.103
*S* = 1.05
4847 reflections
262 parameters
H-atom parameters constrained
Δρ_max_ = 2.07 e Å^−3^
Δρ_min_ = −2.88 e Å^−3^

### 

Data collection: *SMART* (Bruker, 2002 ▶); cell refinement: *SAINT* (Bruker, 2002 ▶); data reduction: *SAINT*; program(s) used to solve structure: *SHELXS97* (Sheldrick, 2008 ▶); program(s) used to refine structure: *SHELXL97* (Sheldrick, 2008 ▶); molecular graphics: *SHELXTL* (Sheldrick, 2008 ▶); software used to prepare material for publication: *SHELXL97*.

## Supplementary Material

Crystal structure: contains datablocks global, I. DOI: 10.1107/S160053681004969X/hb5758sup1.cif

Structure factors: contains datablocks I. DOI: 10.1107/S160053681004969X/hb5758Isup2.hkl

Additional supplementary materials:  crystallographic information; 3D view; checkCIF report

## Figures and Tables

**Table 1:** Selected bond lengths (Å)

Re1—C1	1.887 (6)
Re1—C2	1.921 (5)
Re1—C3	1.890 (5)
Re1—N1	2.228 (4)
Re1—N2	2.169 (4)
Re1—Br1	2.6146 (11)

**Fig. 1.**
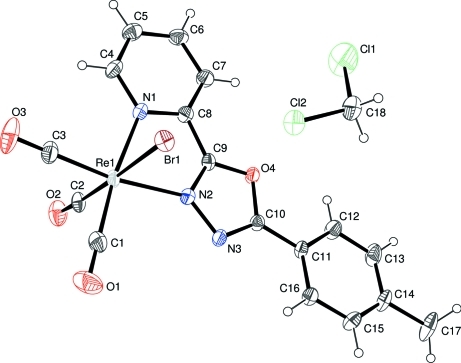
The asymmetric unit of [Re(CO)3(L)Br].CH2Cl2, with displacement ellipsoids drawn at the 30% probability level (arbitrary spheres for the H atoms).

**Fig. 2.**
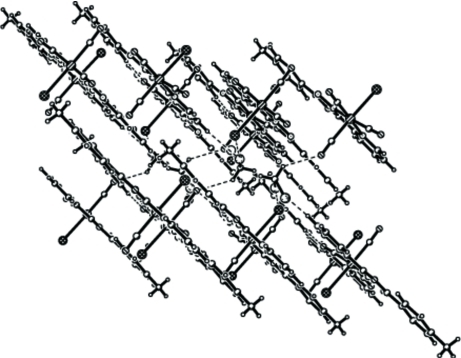
A view of inter-molecular face-to-face stacking structure of (I).

## supplementary crystallographic information

### Comment

As part of our studies of possible phosphorescent materials containing Re(I) (Ley *et al.*, 1997), we have synthesized the title Re(I) complex, (I), which contain the oxadiazole ligand of 2-(pyridin-2-yl)-5-*p*-tolyl-1,3,4-oxadiazole and characterized its structure by single-crystal X-ray diffraction analysis. Its luminescent property will be further studied in the coming research.

As shown in Scheme 1 and Figure 1, [Re(CO)_3_(*L*)Br].CH_2_Cl_2_ is a six-coordinated complex. The coordination geometry at the Re atom is a distorted octahedron with the three carbonyl ligands arranged in a facial fashion. The distances of C(1), C(2), and C(3) to Re(1) are 1.887 (6), 1.921 (5), and 1.890 (5), respectively, and the Re—N bonds distances are 2.228 (4) and 2.169 (4). The bond angles in Table 1 clearly indicate that the CO ligands are linearly coordinated. The bond angles between adjacent CO carbon atoms are 87.8 (3), 89.1 (3) and 89.2 (3) degree, respectively, which is close to 90 degree, but the bond angle between the coordinated nitrogen atoms of ligand is 73.26 (16), which is much less than 90. All other bond distances and angles are comparable to those found for the related Re(I) complexes (Rajendran *et al.*, 2000).

Figure 2 displays inter-molecular face-to-face stacking present in the molecular structure of [Re(CO)_3_(*L*)Br].CH_2_Cl_2_: the 1,3,4-oxadiazole moiety in one molecule is almost parallel to the other one from another complex molecule (the dihedral angle between the two planes is 1.65 degree), and the approximate distance between the two closest atoms (N2—N3) is only 3.349. Thus the complex molecule achieved a bonded dual-molecule structure which is believed a rigid one and will prevent geometric relaxation effectively (Zhang *et al.*, 2009). This kind of rigid structure is expected possessing excellent luminescent properties.

### Experimental

The oxadiazole ligand was synthesized according to the literature method (Demko *et al.*, 2001; Tamoto *et al.*, 1997) with some minor modification.[Re(CO)_3_(*L*)Br] was synthesized according to the following procedure: L1 (0.06 g, 0.210 mmol) and Re(CO)_5_Br (0.08 g, 0.200 mmol) were refluxed in 15 ml of toluene for 6 h. After the mixture was cooled to RT, the solvent was removed in a water bath under reduced pressure. The resulting yellow solid was purified by silica gel column chromatography with acetic acid ethyl ester and dichloromethane (*v*/*v* = 10:1). Yellow blocks of (I) were grown from slow evaporation of a CH_2_Cl_2_ solution.

### Refinement

All H atoms were placed geometrically (N—H = 0.86, C—H = 0.93 Å) and refined as riding with *U*_iso_(H)= 1.2*U*_eq_(carrier).

### Crystal data

**Table:**

[ReBr(C_14_H_11_N_3_O)(CO)_3_]·CH_2_Cl_2_	*F*(000) = 2544
*M**_r_* = 672.32	*D*_x_ = 2.091 Mg m^−^^3^
Monoclinic, *C*2/*c*	Mo *K*α radiation, λ = 0.71073 Å
Hall symbol: -C 2yc	Cell parameters from 4847 reflections
*a* = 12.590 (3) Å	θ = 3.1–24.8°
*b* = 19.621 (4) Å	µ = 7.84 mm^−^^1^
*c* = 17.660 (4) Å	*T* = 293 K
β = 101.77 (3)°	Block, yellow
*V* = 4270.8 (15) Å^3^	0.25 × 0.22 × 0.11 mm
*Z* = 8	

### Data collection

**Table:**

Bruker SMART CCD diffractometer	4847 independent reflections
Radiation source: fine-focus sealed tube	4254 reflections with *I* > 2σ(*I*)
graphite	*R*_int_ = 0.063
phi and ω scans	θ_max_ = 27.5°, θ_min_ = 3.1°
Absorption correction: multi-scan (*SADABS*; Bruker, 2002)	*h* = −14→16
*T*_min_ = 0.149, *T*_max_ = 0.409	*k* = −25→25
20653 measured reflections	*l* = −22→21

### Refinement

**Table:**

Refinement on *F*^2^	Primary atom site location: structure-invariant direct methods
Least-squares matrix: full	Secondary atom site location: difference Fourier map
*R*[*F*^2^ > 2σ(*F*^2^)] = 0.040	Hydrogen site location: inferred from neighbouring sites
*wR*(*F*^2^) = 0.103	H-atom parameters constrained
*S* = 1.05	*w* = 1/[σ^2^(*F*_o_^2^) + (0.0652*P*)^2^ + 4.556*P*] where *P* = (*F*_o_^2^ + 2*F*_c_^2^)/3
4847 reflections	(Δ/σ)_max_ = 0.001
262 parameters	Δρ_max_ = 2.07 e Å^−^^3^
0 restraints	Δρ_min_ = −2.88 e Å^−^^3^

### Special details

**Table:**

Geometry. All e.s.d.'s (except the e.s.d. in the dihedral angle between two l.s. planes) are estimated using the full covariance matrix. The cell e.s.d.'s are taken into account individually in the estimation of e.s.d.'s in distances, angles and torsion angles; correlations between e.s.d.'s in cell parameters are only used when they are defined by crystal symmetry. An approximate (isotropic) treatment of cell e.s.d.'s is used for estimating e.s.d.'s involving l.s. planes.
Refinement. Refinement of *F*^2^ against ALL reflections. The weighted *R*-factor *wR* and goodness of fit *S* are based on *F*^2^, conventional *R*-factors *R* are based on *F*, with *F* set to zero for negative *F*^2^. The threshold expression of *F*^2^ > σ(*F*^2^) is used only for calculating *R*-factors(gt) *etc*. and is not relevant to the choice of reflections for refinement. *R*-factors based on *F*^2^ are statistically about twice as large as those based on *F*, and *R*- factors based on ALL data will be even larger.

### Fractional atomic coordinates and isotropic or equivalent isotropic displacement parameters (Å^2^)

**Table:**

	*x*	*y*	*z*	*U*_iso_*/*U*_eq_	
C1	0.3443 (5)	0.4519 (3)	0.2015 (4)	0.0487 (13)	
C2	0.3684 (5)	0.3907 (2)	0.3391 (3)	0.0382 (11)	
C3	0.1818 (5)	0.4511 (3)	0.2734 (3)	0.0489 (13)	
C4	0.1120 (4)	0.2916 (3)	0.3248 (3)	0.0423 (12)	
H4	0.0859	0.3334	0.3378	0.051*	
C5	0.0700 (4)	0.2331 (3)	0.3493 (3)	0.0472 (13)	
H5	0.0174	0.2355	0.3793	0.057*	
C6	0.1068 (4)	0.1700 (3)	0.3287 (3)	0.0462 (12)	
H6	0.0786	0.1298	0.3444	0.055*	
C7	0.1858 (4)	0.1681 (3)	0.2847 (3)	0.0407 (11)	
H7	0.2118	0.1269	0.2700	0.049*	
C8	0.2249 (3)	0.2291 (3)	0.2634 (2)	0.0329 (10)	
C9	0.3108 (4)	0.2358 (2)	0.2196 (2)	0.0301 (9)	
C10	0.4329 (4)	0.2180 (2)	0.1553 (2)	0.0303 (9)	
C11	0.5036 (4)	0.1774 (2)	0.1171 (2)	0.0312 (9)	
C12	0.5080 (5)	0.1071 (3)	0.1248 (3)	0.0465 (13)	
H12	0.4660	0.0851	0.1550	0.056*	
C13	0.5751 (5)	0.0698 (3)	0.0873 (3)	0.0534 (14)	
H13	0.5771	0.0226	0.0924	0.064*	
C14	0.6396 (5)	0.1012 (3)	0.0422 (3)	0.0490 (14)	
C15	0.6340 (4)	0.1714 (3)	0.0361 (3)	0.0436 (12)	
H15	0.6765	0.1933	0.0062	0.052*	
C16	0.5678 (4)	0.2104 (2)	0.0728 (3)	0.0356 (10)	
H16	0.5662	0.2576	0.0679	0.043*	
C17	0.7105 (7)	0.0591 (4)	0.0005 (4)	0.078 (2)	
H17A	0.7491	0.0888	−0.0277	0.117*	
H17B	0.6659	0.0285	−0.0348	0.117*	
H17C	0.7614	0.0334	0.0375	0.117*	
C18	0.1644 (7)	0.1149 (3)	0.0205 (5)	0.068 (2)	
H18A	0.2392	0.1028	0.0209	0.082*	
H18B	0.1226	0.1075	−0.0314	0.082*	
N1	0.1910 (3)	0.2908 (2)	0.2820 (2)	0.0339 (8)	
N2	0.3461 (3)	0.2940 (2)	0.2029 (2)	0.0327 (8)	
N3	0.4270 (3)	0.2836 (2)	0.1605 (2)	0.0338 (8)	
O1	0.3910 (5)	0.4950 (3)	0.1764 (3)	0.0831 (16)	
O2	0.4232 (4)	0.3958 (2)	0.3981 (3)	0.0620 (12)	
O3	0.1276 (5)	0.4934 (3)	0.2913 (3)	0.094 (2)	
O4	0.3618 (3)	0.18424 (16)	0.19223 (18)	0.0326 (7)	
Br1	0.12760 (5)	0.36874 (3)	0.11151 (3)	0.04326 (14)	
Re1	0.268376 (15)	0.382313 (9)	0.241699 (11)	0.03283 (10)	
Cl1	0.1140 (3)	0.06259 (12)	0.08553 (18)	0.1191 (10)	
Cl2	0.15734 (14)	0.20035 (8)	0.04467 (9)	0.0578 (4)	

### Atomic displacement parameters (Å^2^)

**Table:**

	*U*^11^	*U*^22^	*U*^33^	*U*^12^	*U*^13^	*U*^23^
C1	0.058 (3)	0.034 (3)	0.056 (3)	0.000 (2)	0.015 (3)	−0.004 (2)
C2	0.046 (3)	0.026 (2)	0.040 (3)	0.0082 (19)	0.005 (2)	−0.0054 (18)
C3	0.049 (3)	0.046 (3)	0.051 (3)	0.009 (3)	0.009 (3)	−0.013 (2)
C4	0.037 (3)	0.056 (3)	0.037 (3)	0.008 (2)	0.014 (2)	−0.006 (2)
C5	0.046 (3)	0.058 (3)	0.041 (3)	−0.001 (2)	0.017 (2)	0.004 (2)
C6	0.045 (3)	0.048 (3)	0.049 (3)	−0.009 (2)	0.017 (2)	0.006 (2)
C7	0.046 (3)	0.036 (3)	0.043 (3)	−0.001 (2)	0.014 (2)	0.004 (2)
C8	0.036 (3)	0.034 (3)	0.030 (2)	0.0017 (17)	0.008 (2)	0.0021 (16)
C9	0.037 (2)	0.025 (2)	0.029 (2)	0.0035 (17)	0.0067 (18)	−0.0018 (16)
C10	0.036 (2)	0.030 (2)	0.026 (2)	0.0008 (18)	0.0081 (17)	0.0001 (15)
C11	0.041 (2)	0.029 (2)	0.025 (2)	0.0047 (18)	0.0100 (18)	−0.0011 (15)
C12	0.066 (4)	0.029 (3)	0.052 (3)	0.005 (2)	0.029 (3)	0.004 (2)
C13	0.076 (4)	0.032 (3)	0.057 (3)	0.014 (3)	0.025 (3)	−0.003 (2)
C14	0.059 (3)	0.053 (3)	0.040 (3)	0.017 (3)	0.023 (3)	−0.007 (2)
C15	0.048 (3)	0.052 (3)	0.035 (3)	0.007 (2)	0.019 (2)	0.005 (2)
C16	0.045 (3)	0.031 (2)	0.033 (2)	0.005 (2)	0.013 (2)	0.0034 (18)
C17	0.099 (5)	0.083 (5)	0.061 (4)	0.044 (4)	0.038 (4)	−0.010 (3)
C18	0.074 (5)	0.059 (4)	0.077 (5)	0.004 (3)	0.028 (4)	−0.018 (3)
N1	0.035 (2)	0.036 (2)	0.032 (2)	0.0047 (17)	0.0108 (17)	−0.0038 (16)
N2	0.034 (2)	0.031 (2)	0.035 (2)	0.0007 (16)	0.0111 (17)	−0.0032 (15)
N3	0.040 (2)	0.031 (2)	0.033 (2)	0.0015 (16)	0.0155 (17)	−0.0033 (15)
O1	0.093 (4)	0.054 (3)	0.105 (4)	−0.023 (3)	0.028 (3)	0.019 (3)
O2	0.065 (3)	0.059 (3)	0.054 (3)	0.013 (2)	−0.005 (2)	−0.014 (2)
O3	0.112 (5)	0.079 (4)	0.093 (4)	0.055 (4)	0.025 (3)	−0.022 (3)
O4	0.0407 (17)	0.0271 (16)	0.0328 (16)	0.0012 (13)	0.0144 (14)	−0.0017 (12)
Br1	0.0496 (3)	0.0390 (3)	0.0394 (3)	−0.0001 (2)	0.0049 (2)	0.00145 (19)
Re1	0.03905 (14)	0.02488 (12)	0.03580 (14)	0.00306 (6)	0.01052 (10)	−0.00546 (6)
Cl1	0.176 (3)	0.0556 (13)	0.142 (2)	0.0091 (15)	0.072 (2)	0.0206 (13)
Cl2	0.0680 (9)	0.0531 (9)	0.0511 (8)	0.0077 (7)	0.0089 (7)	0.0014 (6)

### Geometric parameters (Å, °)

**Table:**

Re1—C1	1.887 (6)	C10—N3	1.293 (6)
Re1—C2	1.921 (5)	C10—O4	1.380 (5)
Re1—C3	1.890 (5)	C10—C11	1.459 (6)
Re1—N1	2.228 (4)	C11—C12	1.385 (6)
Re1—N2	2.169 (4)	C11—C16	1.395 (6)
Re1—Br1	2.6146 (11)	C12—C13	1.384 (7)
C1—O1	1.168 (7)	C12—H12	0.9300
C2—O2	1.131 (7)	C13—C14	1.392 (9)
C3—O3	1.159 (7)	C13—H13	0.9300
C4—N1	1.366 (6)	C14—C15	1.383 (8)
C4—C5	1.369 (8)	C14—C17	1.512 (7)
C4—H4	0.9300	C15—C16	1.386 (7)
C5—C6	1.397 (8)	C15—H15	0.9300
C5—H5	0.9300	C16—H16	0.9300
C6—C7	1.381 (7)	C17—H17A	0.9600
C6—H6	0.9300	C17—H17B	0.9600
C7—C8	1.374 (7)	C17—H17C	0.9600
C7—H7	0.9300	C18—Cl2	1.737 (6)
C8—N1	1.346 (6)	C18—Cl1	1.753 (8)
C8—C9	1.459 (7)	C18—H18A	0.9700
C9—N2	1.283 (6)	C18—H18B	0.9700
C9—O4	1.339 (5)	N2—N3	1.396 (5)
			
O1—C1—Re1	179.8 (7)	C15—C16—C11	118.5 (5)
O2—C2—Re1	176.8 (5)	C15—C16—H16	120.7
O3—C3—Re1	178.6 (6)	C11—C16—H16	120.7
N1—C4—C5	122.5 (5)	C14—C17—H17A	109.5
N1—C4—H4	118.8	C14—C17—H17B	109.5
C5—C4—H4	118.8	H17A—C17—H17B	109.5
C4—C5—C6	119.4 (5)	C14—C17—H17C	109.5
C4—C5—H5	120.3	H17A—C17—H17C	109.5
C6—C5—H5	120.3	H17B—C17—H17C	109.5
C7—C6—C5	119.0 (5)	Cl2—C18—Cl1	111.1 (4)
C7—C6—H6	120.5	Cl2—C18—H18A	109.4
C5—C6—H6	120.5	Cl1—C18—H18A	109.4
C8—C7—C6	118.1 (5)	Cl2—C18—H18B	109.4
C8—C7—H7	121.0	Cl1—C18—H18B	109.4
C6—C7—H7	121.0	H18A—C18—H18B	108.0
N1—C8—C7	124.5 (4)	C8—N1—C4	116.6 (4)
N1—C8—C9	110.8 (4)	C8—N1—Re1	117.8 (3)
C7—C8—C9	124.7 (4)	C4—N1—Re1	125.6 (4)
N2—C9—O4	112.1 (4)	C9—N2—N3	108.5 (4)
N2—C9—C8	122.1 (4)	C9—N2—Re1	116.1 (3)
O4—C9—C8	125.9 (4)	N3—N2—Re1	135.4 (3)
N3—C10—O4	113.1 (4)	C10—N3—N2	104.1 (4)
N3—C10—C11	128.8 (4)	C9—O4—C10	102.3 (3)
O4—C10—C11	118.2 (4)	C1—Re1—C3	87.8 (3)
C12—C11—C16	120.1 (4)	C1—Re1—C2	89.1 (3)
C12—C11—C10	121.0 (4)	C3—Re1—C2	89.2 (2)
C16—C11—C10	118.9 (4)	C1—Re1—N2	99.4 (2)
C13—C12—C11	119.7 (5)	C3—Re1—N2	171.4 (2)
C13—C12—H12	120.1	C2—Re1—N2	95.53 (18)
C11—C12—H12	120.1	C1—Re1—N1	172.65 (19)
C12—C13—C14	121.6 (5)	C3—Re1—N1	99.5 (2)
C12—C13—H13	119.2	C2—Re1—N1	91.87 (19)
C14—C13—H13	119.2	N2—Re1—N1	73.26 (16)
C15—C14—C13	117.3 (5)	C1—Re1—Br1	92.72 (19)
C15—C14—C17	122.1 (6)	C3—Re1—Br1	90.28 (18)
C13—C14—C17	120.6 (6)	C2—Re1—Br1	178.05 (17)
C14—C15—C16	122.7 (5)	N2—Re1—Br1	84.79 (11)
C14—C15—H15	118.6	N1—Re1—Br1	86.38 (10)
C16—C15—H15	118.6		

## References

[bb1] Bruker (2002). *SMART*, *SAINT* and *SADABS* Bruker AXS Inc., Madison, Wisconsin, USA.

[bb2] Demko, Z.-P. & Sharpless, K.-B. (2001). *J. Org. Chem.* **66**, 7945–7950. 10.1021/jo010635w 11722189

[bb3] Ley, K.-D., Whittle, C.-E., Bartberger, M.-D. & Schanze, K.-S. (1997). *J. Am. Chem. Soc.* **119**, 3423–3424.

[bb4] Rajendran, T., Manimaran, B., Lee, F.-Y., Lee, G.-H., Peng, S.-M., Wang, C.-C. & Lu, K.-L. (2000). *Inorg. Chem.* **39**, 2016–2017. 10.1021/ic9912474 12526505

[bb5] Sheldrick, G. M. (2008). *Acta Cryst.* A**64**, 112–122. 10.1107/S0108767307043930 18156677

[bb6] Tamoto, N., Adachi, C. & Ngai, K. (1997). *Chem. Mater.* **9**, 1077–1085.

[bb7] Zhang, L.-M., Li, B. & Su, Z.-M. (2009). *Langmuir*, **25**, 2068–2074. 10.1021/la803822s 19170602

